# The survival benefit from surgery on patients with large-cell neuroendocrine carcinoma in the lung: a propensity-score matching study

**DOI:** 10.1186/s13019-023-02314-1

**Published:** 2023-07-05

**Authors:** Hao Jiang, Weixia Xie, Xianpeng Li, Huaying Wang, Wan-Jun Yu, Xiaolu Chen

**Affiliations:** 1 Infectious Department, The Affiliated People’s Hospital of Ningbo University, Yinzhou People’s Hospital, Ningbo, 315040 P. R. China; 2 Hematological Department, The Affiliated People’s Hospital of Ningbo University, Yinzhou People’s Hospital, Ningbo, 315040 P. R. China; 3 Department of Respiratory and Critical Care, Yinzhou People’s Hospital, The Affiliated People’s Hospital of Ningbo University, Ningbo, 315040 P. R. China

**Keywords:** Large-cell neuroendocrine carcinoma, Survival, Surgery, Propensity-score matching

## Abstract

**Purpose:**

This study aimed to investigate the prognostic significance of surgery in large-cell neuroendocrine carcinoma (LCNC) patients.

**Methods:**

A total of 453 patients from the Surveillance, Epidemiology, and End Results database diagnosed with stage T1-4N0-2M0 LCNC from 2010 to 2015 were analyzed. The propensity-score matching analysis with a ratio of 1:1 was used to minimize the bias effect of other clinical characteristics, and 77 pairs of patients’ data were performed for subsequent statistical analysis. The Cox proportional hazards model, Kaplan-Meier analysis, and Log-rank test were used in the present study. The primary observational endpoint was cancer-specific survival (CSS).

**Results:**

The 1-year, 3-year, and 5-year CSS rates were 60.0%, 45.0%, and 42.0% in those 453 LCNC patients. Compared with patients who underwent surgical resection, patients without surgery had a lower 5-year CSS rate (18.0% vs. 52.0%, *P* < 0.001). After analyses of multivariable Cox regression, chemotherapy, T stage, N stage, and surgery were identified as independent prognostic indicators (all *P* < 0.05). In the cohort of old patients, the median survival time was longer in cases after surgery than those without surgery (13.0 months vs. NA, *P* < 0.001). Besides, in patients with different clinical characteristics, the receiving surgery was a protective prognostic factor (all hazard ratio < 1, all *P* < 0.05). In addition, for the cohort with stage T1-2N0-2M0, patients after the operation had more improved outcomes than patients without surgery (*P* < 0.001).

**Conclusions:**

We proposed that the surgery could improve the survival outcomes of LCNC patients with stage T1-4N0-2M0. Moreover, old patients could benefit from surgery.

## Introduction

Although lung cancer has shown a decline in incidence worldwide in recent years, its mortality rate remains at the top of the malignant disease spectrum [1]. Among lung cancers, neuroendocrine carcinoma is not a common pathological type, mainly including small-cell carcinoma, combined small-cell carcinoma, large-cell carcinoma, and large-cell neuroendocrine carcinoma (LCNC) [2, 3]. As for LCNC, its treatment is now considered to be closer to that of small-cell carcinoma [4]. Surgical resection has been shown to be effective for limited-stage small-cell lung cancer, but the role of surgery for large-cell neuroendocrine carcinoma still needs further clarification [5, 6].

Old age is a risk factor for prognoses of malignancies, confirmed by other studies [6–9]. Besides, previous reports showed that the rate of perioperative complications in old patients was higher than in young patients [10, 11]. Therefore, exploring the significance of receiving surgery in old patients is valuable. However, the study on illuminating the association between surgery and old LCNC patients is currently lacking. Thus, this study aimed to uncover surgery’s role in the LCNC and old patients.

## Methods

### Patients

**Fig. 1:**
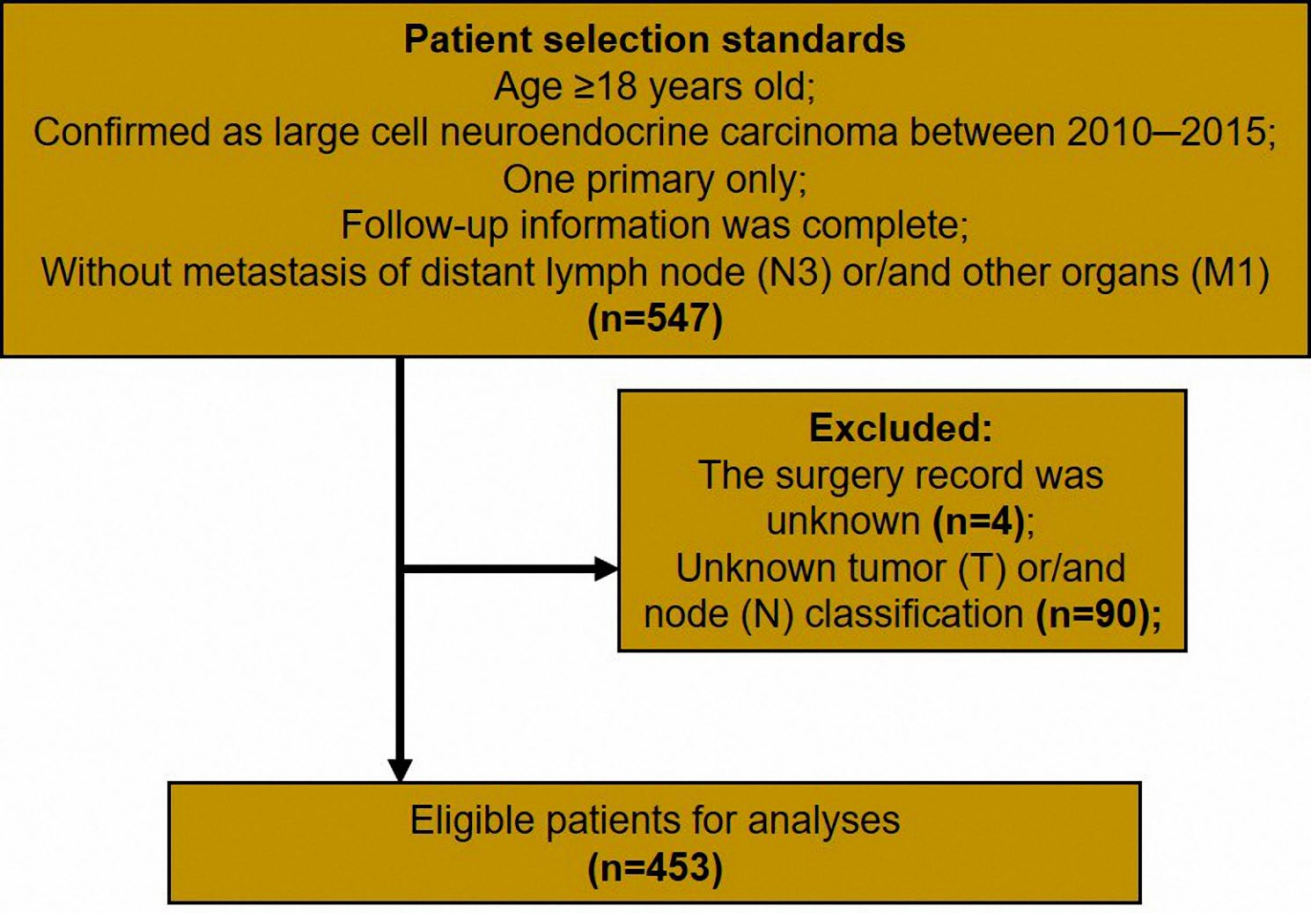
The process of patient selection in this study

The ethics committee of The Affiliated People’s Hospital of Ningbo University approved this study and considered this study exempt from ethical review because existing data with patient de-identifiers were used. This study included patients from the Surveillance, Epidemiology, and End Results (SEER) database who met the following criteria: (1) age older than 17 years; (2) pathologically confirmed LCNC (International Classification of Disease for Oncology third edition code: 8013/3) between 2010─2015 [12]; (3) one primary only; (4) follow-up information was complete; (5) without distant metastasis of lymph node (N3) or/ and other organs (M1). In addition, patients who met the following standards were excluded from this study: (1) patients who had unknown surgery records; (2) patients who had an unknown tumor (T) or/ and node (N) classification. Details of the patient selection standards are shown in Fig. 1.

### Follow-up

Cancer-specific survival (CSS) is the duration from the diagnosis to death caused by lung cancer, which was regarded as our observational endpoint. The eligible patients had a clear survival time and survival status.

### Statistical analysis

Statistical analysis was performed using SPSS Statistics 25.0 software (IBM SPSS, Inc, Armonk, IL, USA). The Chi-square test was used to determine the associations between surgery and other clinical features. Univariable and multivariable proportional hazard regression models were used to calculate the hazard ratio (HR) and 95% confidence interval (CI) on cancer-specific mortality. Kaplan–Meier analysis and log-rank tests were performed to draw and compare survival curves between the groups. Statistical tests were considered statistically significant with a two-sided *P* value < 0.05.

### Propensity-score matching

To minimize the bias effect of other clinical variables, we used sex, age, marital status, tumor location, laterality, N stage, T stage, grade, radiotherapy, and chemotherapy as matching variables [6, 13]. After propensity-score matching (PSM) with a match tolerance of 0.01, the equilibrium test between the groups was checked via the χ2 test. The ratio of PSM was 1:1.

## Results

### Patient characteristics

In the present study, 453 patients were eligible for main analyses. The man patients outnumbered woman patients (52.5% vs. 47.5%). Most patients were over 65 years (n = 233, 51.4%). Two hundred ninety-nine patients received surgery, and the majority of patients who underwent surgery were in the T1 or N0 stage. Overall, significant differences in all clinicopathological variables except sex and age (Table 1, all *P* < 0.05) before PSM. To better compare the two groups, using PSM at a ratio of 1:1, 154 patients remained in the group. Except for marital status (*P* < 0.001), there were no significant differences in the clinicopathological patient characteristics between the two groups after PSM, as shown in Table 1.

**Table 1:** The baseline characteristics of large-cell neuroendocrine carcinoma patients

		Surgery			Surgery (PSM)		
Variables	All n = 453 (%)	No n = 154 (%)	Yes n = 299 (%)	*P*-value	No n = 77 (%)	Yes n = 77 (%)	*P*-value
Sex				0.226			1.00
Male	238 (52.5)	87 (56.5)	151 (50.5)		44 (57.1)	44 (57.1)	
Female	215 (47.5)	67 (43.5)	148 (49.5)		33 (42.9)	33 (42.9)	
Age (year)				0.052			0.871
≤ 65	220 (48.6)	65 (42.2)	155 (51.8)		34 (44.2)	35 (45.5)	
> 65	233 (51.4)	89 (57.8)	144 (48.2)		43 (55.8)	42 (54.5)	
Race				0.019			0.841
White	363 (80.1)	114 (74.0)	249 (83.3)		61 (79.2)	62 (80.5)	
Other/ unknown	90 (19.9)	40 (26.0)	50 (16.7)		16 (20.8)	15 (19.5)	
Marital status				0.027			< 0.001^*^
Unmarried	211 (46.6)	85 (55.2)	218 (42.1)		48 (62.3)	23 (29.9)	
Married	211 (48.8)	62 (40.3)	255 (53.2)		26 (33.8)	50 (64.9)	
Unknown	21 (4.6)	7 (4.5)	20 (4.7)		3 (3.9)	4 (5.2)	
Location				< 0.001			0.297
Upper	302 (66.7)	102 (66.2)	200 (66.9)		50 (64.9)	49 (64.3)	
Lower	114 (25.2)	29 (18.8)	85 (28.4)		16 (20.8)	22 (24.7)	
Other/ unknown	37 (8.1)	23 (15.0)	29 (4.7)		11 (14.3)	17 (11.0)	
Laterality				0.043^*^			0.513
Left	199 (43.9)	58 (37.7)	141 (47.2)		30 (39.0)	34 (44.2)	
Right	253 (55.8)	95 (61.7)	158 (52.8)		47 (61.0)	43 (55.8)	
Other/ unknown	1 (0.3)	1 (0.6)	0 (0.0)		-	-	
T stage				< 0.001			0.668
T1	186 (41.1)	50 (32.5)	136 (45.5)		33 (42.9)	28 (36.4)	
T2	145 (32.0)	39 (25.3)	106 (35.5)		22 (28.6)	26 (33.8)	
T3	47 (10.4)	15 (9.7)	32 (10.7)		5 (6.5)	8 (10.4)	
T4	75 (16.5)	50 (32.5)	25 (8.3)		17 (22.0)	15 (19.4)	
N stage				< 0.001			0.144
N0	286 (63.1)	67 (43.5)	219 (73.2)		44 (57.1)	44 (57.1)	
N1	54 (11.9)	7 (4.5)	47 (15.7)		5 (6.5)	12 (15.6)	
N2	113 (25.0)	80 (52.0)	33 (11.1)		28 (36.4)	21 (27.3)	
Grade				< 0.001			0.211^*^
I─II	11 (2.4)	3 (1.9)	8 (2.7)		3 (3.8)	2 (2.6)	
III	231 (51.0)	58 (37.7)	173 (57.9)		36 (46.8)	29 (37.6)	
IV	65 (14.3)	67 (7.1)	54 (18.1)		5 (6.5)	13 (16.9)	
Unknown	146 (32.3)	163 (53.3)	64 (21.3)		33 (42.9)	33 (42.9)	
Radiotherapy				< 0.001			0.306^*^
No	295 (65.1)	45 (29.2)	250 (83.6)		35 (45.5)	38 (49.4)	
Yes	151 (33.3)	106 (68.8)	45 (15.1)		41 (53.2)	35 (45.5)	
Unknown	7 (1.6)	3 (2.0)	4 (1.4)		1 (1.3)	4 (5.1)	
Chemotherapy				< 0.001			0.747
No	237 (52.3)	55 (35.7)	182 (60.9)		38 (49.4)	36 (46.8)	
Yes	216 (47.7)	99 (64.3)	117 (39.1)		39 (50.6)	41 (53.2)	

PSM: propensity-score matching, Fisher’s exact test calculated the *P*-value of comparison to laterality^*^, and the Chi-square test calculated others

### Survival outcomes

**Fig. 2:**
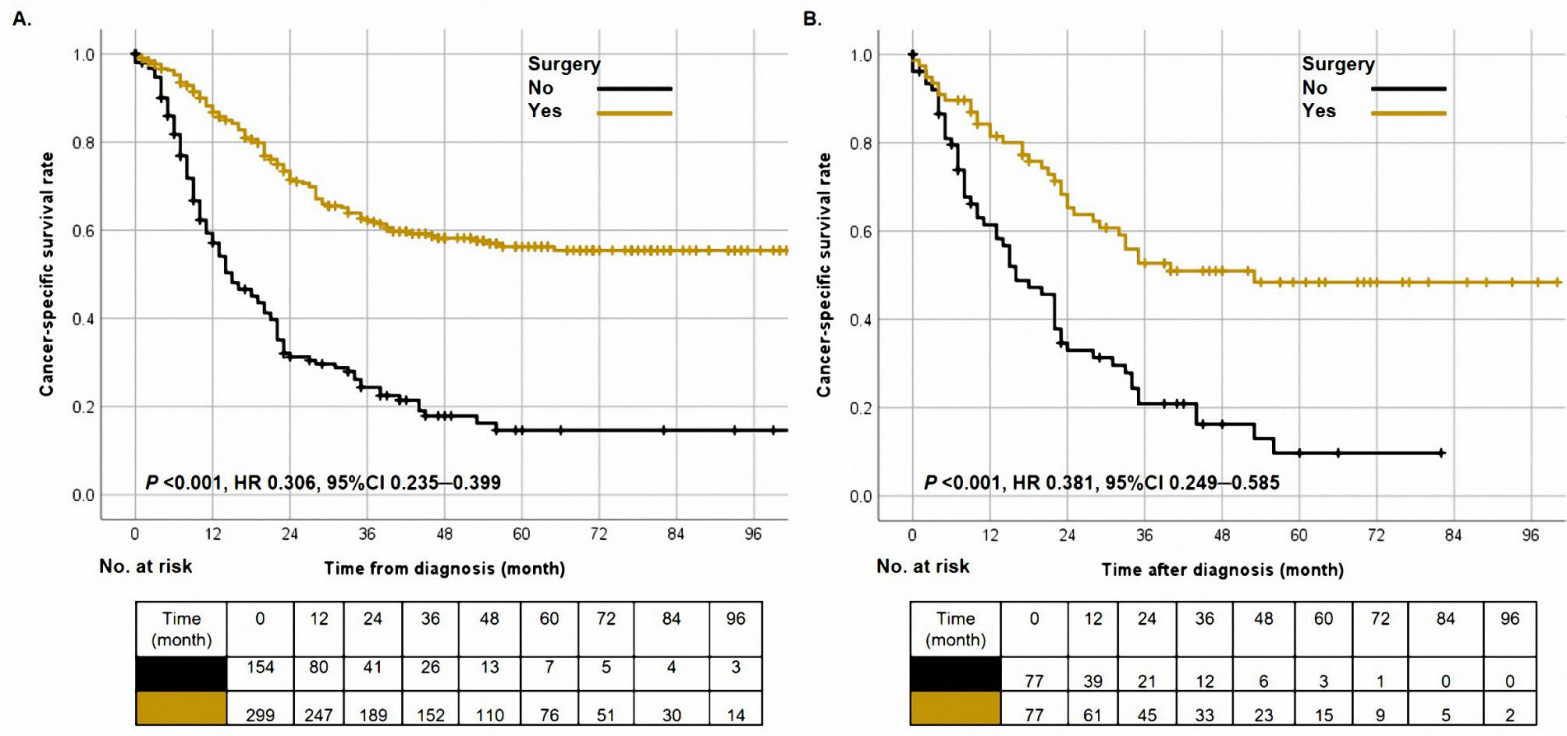
The cancer-specific survival curves in all patients **(A)** and matched cohort **(B)** based on surgery (no vs. yes)

The median survival time of this cohort was 24.0 months (range 0─106.0 months). The 1-year, 3-year, and 5-year CSS rates were 60.0%, 45.0%, and 42.0% in those 453 LCNC patients, respectively. Besides, the cases with surgery had better survival than cases without surgery (Fig. 2A, Log-rank *P* < 0.001). Compared with patients who underwent surgical resection, patients without surgery had a lower 5-year CSS rate (18.0% vs. 52.0%). Moreover, in the matched cohort, surgery’s role in the prognosis was encouraging. The surgery improved the 5-year CSS rate for this group of patients by 27% (Fig. 2B, Log-rank *P* < 0.001). After analyses of univariable and multivariable Cox regression, chemotherapy, T stage, N stage, and surgery (adjusted HR 0.315, 95%CI 0.218─0.457, *P* < 0.001) were identified as independent prognostic indicators (Table 2, all *P* < 0.05).

**Table 2:** Cox regression analyses for cancer-specific mortality of patients

	Univariable analysis			Multivariable analysis		
Variables	HR	95% CI	*P*-value	HR	95% CI	*P*-value
Sex						
Male	1	reference		1	reference	
Female	0.757	0.582─0.985	0.039	0.911	0.692─1.200	0.507
Age (year)						
≤ 65	1	reference		1	reference	
> 65	1.275	0.981─1.657	0.069	1.232	0.940─1.615	0.130
Race						
White	1	reference				
Other/ unknown	1.301	0.956─1.770	0.094			
Marital status						
Unmarried	1	reference				
Married	0.978	0.750─1.276	0.870			
Unknown	0.842	0.410─1.727	0.638			
Location						
Upper	1	reference		1	reference	
Lower	1.169	0.860─1.589	0.319	1.318	0.963─1.804	0.085
Other/ unknown	2.049	1.338─3.136	0.001	1.491	0.954─2.331	0.080
Laterality						
Left	1	reference				
Right	1.114	0.856─1.450	0.421			
Other/ unknown	5.907	0.816─42.75	0.079			
T stage						
T1	1	reference		1	reference	
T2	1.464	1.058─2.026	0.021	1.506	1.080─2.101	0.016
T3	1.991	1.289─3.075	0.002	1.997	1.269─3.142	0.003
T4	2.812	1.969─4.016	< 0.001	2.135	1.428─3.194	< 0.001
N stage						
N0	1	reference		1	reference	
N1	2.304	1.592─3.334	< 0.001	2.846	1.935─4.186	< 0.001
N2	2.908	2.168─3.900	< 0.001	2.488	1.742─3.553	< 0.001
Grade						
I─II	1	reference				
III	1.085	0.443─2.656	0.859			
IV	0.780	0.300─2.026	0.610			
Unknown	1.441	0.583─3.560	0.429			
Surgery						
No	1	reference		1	reference	
Yes	0.306	0.235─0.399	< 0.001	0.315	0.218─0.457	< 0.001
Radiotherapy						
No	1	reference		1	reference	
Yes	1.947	1.492─2.541	< 0.001	0.847	0.587─1.223	0.377
Unknown	1.521	0.561─4.121	0.410	0.668	0.237─1.881	0.445
Chemotherapy						
No	1	reference		1	reference	
Yes	0.306	0.235─0.399	< 0.001	0.532	0.384─0.739	< 0.001

The method of Cox regression is “Enter selection”

**Fig. 3:**
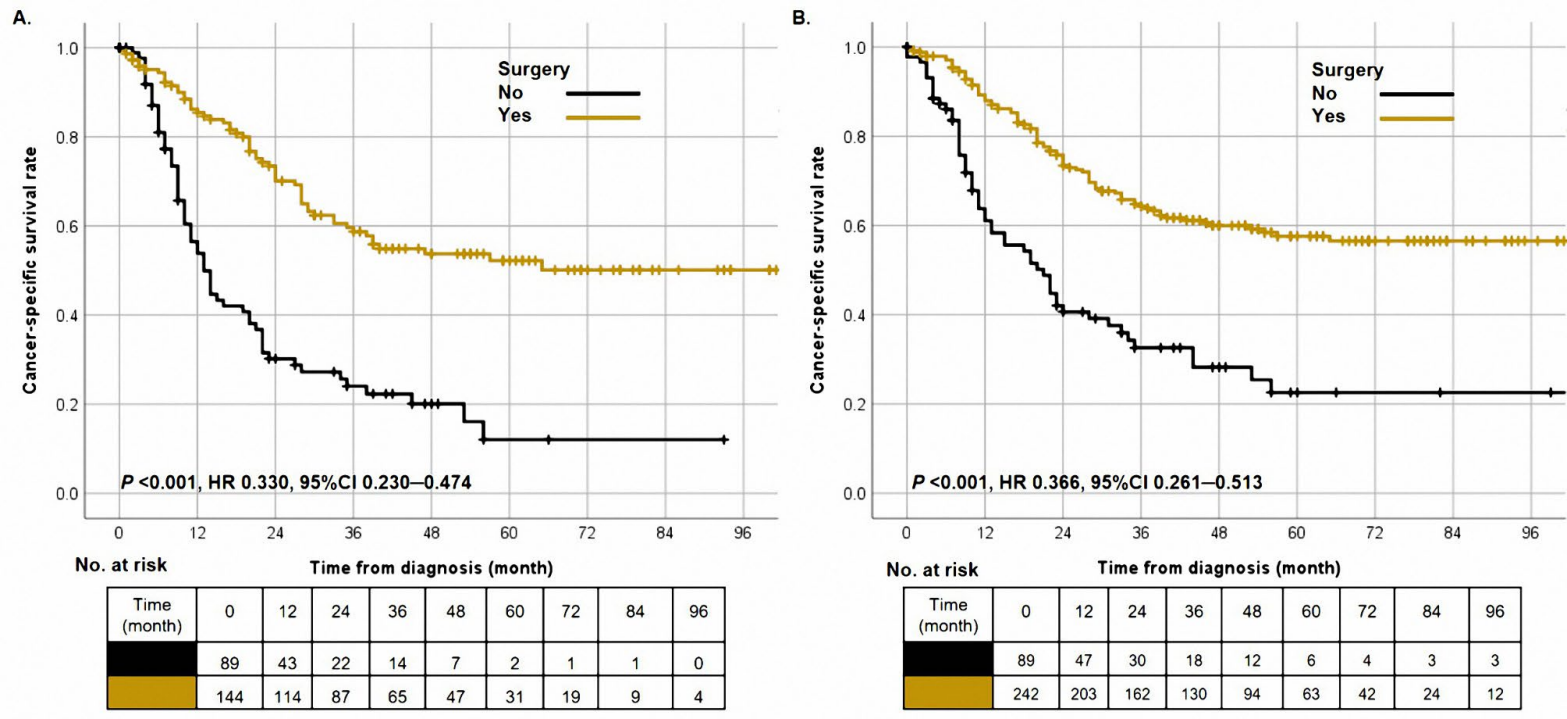
The cancer-specific survival curves in old patients **(A)** and patients with stage T1-2N0-2M0 **(B)** based on surgery (no vs. yes)

A sub-group analysis was performed further to explore surgery’s prognostic significance in old LCNC patients. In the cohort of old patients, the 1-year, 3-year, and 5-year CSS rates were 58.0%, 41.0%, and 37.0%, respectively. In addition, the median survival time was longer in cases after surgery than those without surgery (13.0 months vs. NA, Fig. 3A, P < 0.001). Besides, we explored the prognostic characteristics of old LCNC patients. Surgery, T stage, N stage, and chemotherapy were confirmed as independent prognostic factors (Table 3, all *P* < 0.05).

**Table 3:** Cox regression analyses for cancer-specific mortality of old patients

	Univariable analysis			Multivariable analysis		
Variables	HR	95% CI	*P*-value	HR	95% CI	*P*-value
Sex						
Male	1	reference		1	reference	
Female	0.754	0.558─1.077	0.121	0.880	0.603─1.285	0.508
Race						
White	1	reference				
Other/ unknown	1.448	0.912─2.298	0.117			
Marital status						
Unmarried	1	reference				
Married	1.043	0.728─1.493	0.820			
Unknown	0.608	0.148─2.493	0.490			
Location						
Upper	1	reference		1	reference	
Lower	1.290	0.863─1.928	0.319	1.426	0.942─2.159	0.093
Other/ unknown	1.994	1.082─3.677	0.027	1.511	0.779─2.931	0.222
Laterality						
Left	1	reference				
Right	0.956	0.670─1.364	0.805			
T stage						
T1	1	reference		1	reference	
T2	2.069	1.361─3.144	0.001	2.076	1.332─3.236	0.001
T3	2.082	1.097─3.953	0.025	1.778	0.904─3.498	0.096
T4	2.808	1.661─4.749	< 0.001	2.009	1.096─3.681	0.024
N stage						
N0	1	reference		1	reference	
N1	1.812	0.978─3.358	0.059	1.787	1.914─3.494	0.090
N2	2.883	1.955─4.251	< 0.001	2.726	1.696─4.382	< 0.001
Grade						
I─II	1	reference				
III	1.040	0.325─3.326	0.948			
IV	0.775	0.226─2.659	0.685			
Unknown	1.535	0.478─4.929	0.472			
Surgery						
No	1	reference		1	reference	
Yes	0.330	0.230─0.474	< 0.001	0.278	0.169─0.457	< 0.001
Radiotherapy						
No	1	reference		1	reference	
Yes	1.405	0.970─2.035	0.072	0.623	0.377─1.027	0.064
Unknown	1.482	0.466─4.709	0.505	1.182	0.352─3.975	0.787
Chemotherapy						
No	1	reference		1	reference	
Yes	1.182	0.826─1.693	0.361	0.444	0.281─0.703	0.001

The method of Cox regression is “Enter selection”

**Table 4:** Univariable Cox regression analysis for cancer-specific mortality according to surgery (no vs. yes)

	Univariable analysis		
Sub-group	HR	95% CI	*P*-value
Sex			
Male	0.324	0.228─0.462	< 0.001
Female	0.293	0.196─0.438	< 0.001
Age (year)			
≤ 65	0.287	0.194─0.424	< 0.001
T stage			
T1	0.275	0.172─0.438	< 0.001
T2	0.505	0.308─0.827	0.007
T3	0.323	0.150─0.696	0.004
T4	0.250	0.120─0.521	< 0.001
N stage			
N0	0.280	0.190─0.412	< 0.001
N1	0.402	0.173─0.935	0.034
N2	0.451	0.262─0.777	0.004
Radiotherapy			
No	0.148	0.098─0.224	< 0.001
Yes	0.622	0.398─0.974	0.038
Chemotherapy			
No	0.232	0.154─0.348	< 0.001
Yes	0.361	0.250─0.521	< 0.001

The method of Cox regression is “Enter selection”

Then, we further investigated the role of surgery on patients with different clinical characteristics. In the other groups, the receiving surgery was a protective prognostic factor (Table 4, all HR < 1, all *P* < 0.05). In addition, for the cohort with stage T1-2N0-2M0, patients after the operation had more improved outcomes than patients without surgery (Fig. 3B, P < 0.001).

## Discussion

The discussion on pulmonary neuroendocrine carcinoma has focused mainly on small-cell carcinoma. For LCNC, studies on surgery affecting survival still need to be completed. Thus, in the present study, we used a database of a large sample size to explore the association between surgery and prognosis in LCNC patients. The univariable and multivariable Cox regression analyses were used to identify the factor affecting prognosis. In addition, we used PSM analysis to confirm that receiving surgery could improve the survival outcomes of LCNC patients and reduce bias from other clinical and pathological features. Sub-group analyses were performed further to explore the relationship between surgery and other characteristics. Besides, we found that patients with operation had better survival than patients without surgery in cohorts with different clinical and pathological factors, such as old age, stage T1-4N0-2M0, and chemotherapy. Therefore, we propose that LCNC patients should undergo surgery if there are no contraindications to surgery.

As a classical treatment approach, surgical resection has been proven to be associated with extended survival in patients with neuroendocrine carcinoma [6, 14, 15]. Previous studies focused on small-cell lung cancer and reported that patients with stage I-IIA were more appropriate to receive surgery than those with stage IIb-III [5, 16]. Recently, a study from *Gao et al.* suggested that patients with stage III who underwent surgery had potential survival benefits [17]. Accordingly, those results indicated that the staging status of patients with neuroendocrine carcinoma suitable for the surgery needed to be further determined. In the present study, we investigated the prognostic significance of surgery in LCNC patients with stage T1-2N0-2M0 and found that those patients with operation had improved survival outcomes. Besides, surgery could provide an improved prognosis in different statuses of the N stage, including N0, N1, and N2. Therefore, we suggest that the surgery is a worthy treatment approach for LCNC patients with clinical stage T1-4N0-2M0 to consider.

In addition, old age was not a prognostic risk factor statistically in this study, unlike other studies [18, 19]. The reason for this phenomenon might be that the sample size was small, and the cutoff point of age was different. For elderly lung cancer patients, it has been controversial whether surgery is performed or not [20–22]. In this study, we performed a sub-group analysis in patients with ages > 65 years and found that old patients could reach a longer median survival time after surgery. Therefore, a study by *Rogers SO Jr et al.* revealed that age was not a limiting factor for surgery [20]. Whether the surgery is performed or not depends mainly on the patient’s concomitant disease, lung function, and other contraindications to surgery. With the popularity of video-assisted thoracoscopic surgery, patients’ postoperative recovery period has been shortened, and patients suffer less pain and postoperative complications [23]. In addition, a recent report uncovered that overall survival and recurrence-free survival in patients with video-assisted thoracoscopic surgery was comparable to cases using traditional thoracotomy [23]. Those findings provided for the surgical treatment of elderly LCNC patients.

Some inevitable limitations should be indicated in this study. First, the clinical and pathological characteristics analyzed in this study are not comprehensive because the quantity of data released in SEER is limited; thus, the potential bias or error could be caused although the PSM was performed. Second, tumor markers and molecular tests were not included in our study. Third, the study was retrospective though it had a large sample size. Thus, we still need prospective studies to confirm those findings.

## Conclusions

We proposed that the surgery could improve the survival outcomes of LCNC patients with stage T1-4N0-2M0. Moreover, old patients could benefit from surgery.

## Data Availability

Any researchers interested in this study could contact us to request the data.

## Abbreviations

LCNC: Large-cell neuroendocrine carcinoma
SEER: Surveillance, Epidemiology, and End Results
CSS: Cancer-specific survival
T: Tumor
N: Node
M: Metastasis
HR: Hazard ratios
CI: Confidence intervals
PSM: Propensity-score matching
